# Effects of Forging Temperature and Micro-Arc Coatings on the Static/Stress Corrosion Resistance of AZ80 Magnesium Alloy

**DOI:** 10.3390/ma18112590

**Published:** 2025-06-01

**Authors:** Yuna Xue, Jie Zhang, Yi Shen, Yongpeng Qiao, Sheji Luo, Di Wang

**Affiliations:** 1 School of Materials Science and Engineering, Xi’an Shiyou University, Xi’an 710065, China; zj11262025@163.com (J.Z.); 371548234@163.com (Y.S.); sjluo@xsyu.edu.cn (S.L.); 2 Xi’an Flit Heat Treatment Co., Ltd., Xi’an 710065, China; vincoq@163.com; 3 Shaanxi Key Laboratory of Surface Engineering and Remanufacturing, Xi’an University, Xi’an 710065, China; wangd@xawl.edu.cn

**Keywords:** wrought magnesium alloy, micro-arc oxidation, micro-arc composite, static/stress corrosion characterization

## Abstract

To enhance the surface protection of exposed moving parts made from magnesium alloys, this study focuses on developing high-performance micro-arc composite (MCC) coatings on AZ80 wrought magnesium alloy substrate. AZ80 alloys were fabricated through forging at different temperatures (250 °C, 350 °C, and 450 °C) to investigate the influence of thermal deformation on substrate properties. Subsequently, micro-arc oxidation (MAO) coatings and MCC coatings were applied to the forged alloys. Comprehensive analyses—including microstructural characterization, salt spray corrosion tests, and stress corrosion cracking (SCC) evaluations—were conducted under both static and stress conditions. Among the forging temperatures, 250 °C produced substrates with refined grains and a favorable distribution of β-Mg_17_Al_12_ precipitates, resulting in improved baseline corrosion resistance. MAO coatings offered moderate protection, primarily delaying corrosion initiation and crack propagation under stress environments. Building upon this foundation, MCC coatings—fabricated by electrostatic spraying to form an inner-embedded and outer-wrapped structure over the MAO layer—demonstrated significantly superior protective performance. Under both static and stress corrosion scenarios, the MCC coatings effectively suppressed SCC initiation and progression, highlighting their potential for robust surface protection in demanding service environments.

## 1. Introduction

Weight reduction has long been a critical focus in fields such as aerospace and transportation, playing a pivotal role in the design of transportation equipment that prioritizes lower emissions and enhanced fuel efficiency. This aligns closely with the global objectives of achieving “carbon peak and carbon neutrality”. When integrated with an appropriately sized power-train, light-weighting can significantly improve vehicle fuel economy, with studies indicating that a 10% reduction in weight can lead to a 7% decrease in fuel consumption. Magnesium alloys, owing to their low density, high specific strength, and specific modulus, as well as their excellent processing properties, are considered the ideal choice for engineering applications. They are anticipated to emerge as one of the primary materials for structural light-weighting [1,2,3,4]. However, magnesium alloys are characterized by high electrochemical activity, poor corrosion resistance, and limited ductility. This is particularly concerning when they are employed as structural components, as they are often exposed to the combined effects of mechanical loads and corrosive environments in real-world applications. Such conditions make them susceptible to stress corrosion cracking (SCC) and premature failure, which significantly restricts their broader application [5,6,7,8,9]. Magnesium alloys demonstrate a pronounced tendency for stress corrosion cracking in environments such as industrial settings, marine atmospheres, sodium chloride solutions, and potassium chromate solutions [10]. Stress corrosion failure is particularly hazardous due to its concealed nature and severe consequences; it can result in brittle fractures even under relatively low stress levels (well below the yield strength), thereby posing a substantial risk of catastrophic failures [11].

Currently, extensive efforts have been dedicated to improving the corrosion resistance of magnesium alloys, ranging from enhancing the intrinsic properties of the magnesium alloy substrate to the development of advanced surface protective coatings [12,13,14,15,16]. In terms of the magnesium alloy substrate, primary strategies include the incorporation of alloying elements and the application of various forming techniques. Notably, wrought magnesium alloys have emerged as a significant advancement. Compared to their cast counterparts, wrought magnesium alloys demonstrate superior mechanical properties, higher strength, and the ability to be fabricated into complex geometries. As high-strength wrought magnesium alloys are increasingly utilized in structural applications, addressing their relatively poor corrosion resistance has become an essential and pressing area of research [17,18]. Gryguc et al. [19,20] demonstrated in their research that forging AZ80 magnesium alloy at certain temperatures not only enhances its corrosion resistance but also improves its mechanical performance.

Surface coatings are widely regarded as one of the most cost-effective and efficient approaches to improving the corrosion resistance of magnesium alloys. Xiong et al. [21] discovered that a composite coating formed by laser shock peening combined with micro-arc oxidation significantly enhances the stress corrosion cracking (SCC) resistance of AZ80 alloy. Research by Srinivasan et al. [22] indicated that plasma electrolytic oxidation coatings markedly improve the static corrosion performance of AZ61 wrought magnesium alloys, although they did not observe a significant improvement in SCC resistance. Research on the stress corrosion resistance of surface coatings on magnesium alloys is relatively scarce. Consequently, there is a critical need to design and develop innovative composite coatings that not only enhance static corrosion resistance but also improve stress corrosion cracking performance under the combined effects of mechanical stress and environmental factors.

The porous structure of the micro-arc oxidation coating (MAO) makes it highly compatible with various materials, enabling the formation of composite coatings with multifunctional properties. This highlights the versatility of the ceramic oxide layer as an effective “transition treatment layer”. Micro-arc composite coating (MCC) technology integrates micro-arc oxidation without pretreatment and organic coating techniques, such as electro-induced influx or electrostatic spraying, which are known for their excellent static protection performance. This approach creates a micro-arc composite gradient coating characterized by internal embedding and external encapsulation. MCC technology is recognized as an environmentally friendly surface treatment method, offering advantages such as a streamlined process, low pollution emissions, and high efficiency [9,23]. By applying this technology, high-performance and multifunctional micro-arc composite coatings can be fabricated on magnesium alloy surfaces. These coatings combine the benefits of a simple process, environmental sustainability, zero emissions, high treatment efficiency, superior overall coating performance, and broad material adaptability.

In this study, the preparation of magnesium alloy automotive control arms serves as the research context. Wrought magnesium alloys were formed by forging AZ80 extruded magnesium alloy at three different temperatures (250 °C, 350 °C, and 450 °C). Microstructural characterization is subsequently performed to analyze the material properties. The influence of factors such as grain size, second-phase particles, and impurities on the corrosion behavior of magnesium alloys is systematically investigated. Furthermore, micro-arc oxidation (MAO) coatings and micro-arc composite coatings (MCC) are fabricated and subjected to static corrosion tests and slow strain rate tensile tests. This research aims to explore the static and stress corrosion behavior and underlying mechanisms of these coatings. The results provide a deeper understanding of stress-corrosion behavior in wrought magnesium alloys. The development of effective surface protection strategies against both static and stress corrosion can promote the application of high-strength magnesium alloy structural materials.

## 2. Materials and Methods

### 2.1. Experimental Materials and Specimen Preparation

The AZ80 magnesium alloy demonstrates exceptional suitability for forging applications, emerging as a highly promising material for manufacturing lightweight automotive components through closed-die forging processes [19,24,25]. The alloy was supplied in extruded rod form with dimensions of 63.5 mm in diameter and 1000 mm in length. Its chemical composition comprises: 8.2% Al, 0.42% Zn, 0.31% Mn, 0.10% Si, 0.05% Cu, 0.005% Ni, and 0.005% Fe, with the balance being Mg.

**Figure 1:**
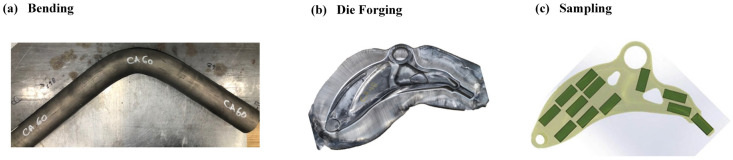
The plan view of the extruded billet during various stages of the forging operation for achieving the final component (before flash trimming) and sampling map.

Figure 1 illustrates the primary forging process workflow. The initial billets were sectioned to 680 mm lengths (Figure 1a). During the process, the billets underwent bending to 108 ° using a mandrel bender to approximate the component’s final geometry and curvature (Figure 1b, Step 2). Subsequent forging operations were conducted on a 1500-ton hydraulic press equipped with heated upper and lower dies featuring intricate internal geometries corresponding to an automotive suspension’s lower control arm (Figure 1c). Prior to forging, the raw material underwent furnace heating to predetermined temperatures (250 °C, 350 °C, and 450 °C) for a sufficient duration to ensure thermal uniformity. The heated billets were then transferred to isotherm molds, where the hydraulic press applied pressure at a constant displacement rate of 20 mm/s to complete the forging operation in a single step, achieving the target component geometry. Notably, the forging direction was maintained perpendicular to the original extrusion axis. Graphite lubricant was employed throughout the process to reduce frictional effects. Following forging, the specimens were extracted from the molds and allowed to air-cool at ambient temperature without subsequent heat treatment.

Specimens were extracted from the AZ80 extruded rod along its extrusion direction. The sampling locations of the forged magnesium alloys processed at different temperatures are illustrated in Figure 1c. All specimens were machined to dimensions of 50 mm × 25 mm × 3 mm for subsequent corrosion testing.

To facilitate comprehensive microstructural characterization of AZ80 extruded and forged magnesium alloys, a standardized metallographic preparation protocol must be rigorously followed. The procedure commences with cold-mounting specimens in epoxy resin to ensure structural integrity during subsequent processing. Following mounting, specimens undergo systematic mechanical grinding using progressively finer silicon carbide abrasive papers ranging from 400 to 1200 grit, achieving surface polarization through controlled material removal. Subsequent fine polishing employs diamond suspension to obtain a mirror-finish surface essential for microscopic analysis.

The chemical etching process utilized an acetic acid-piratical etchant formulated by combining 70 mL anhydrous ethanol (95% purity), 10 mL glacial acetic acid, 4.2 g crystalline picric acid, and 10 mL deionized water. This specific ratio of etching solution can produce a moderate corrosion effect on the surface of the magnesium alloy, enhancing the contrast between different phases and thus more clearly revealing the microstructure of the alloy. After etching, the specimens are immediately washed in alcohol to remove the residual etchant, dried with air, and observed under an optical microscope. For electron backscatter diffraction (EBSD) analysis of AZ80 magnesium alloy, a dual-stage preparation methodology combining electrochemical polishing and precision ion milling was implemented to achieve stress-free specimen surfaces. This primary treatment effectively removed mechanical deformation layers while preserving crystallographic integrity. Subsequent ion beam thinning (4 kV Ar^+^ at 4° incidence angle) was performed to eliminate residual surface oxides and achieve the sub-micron surface finish required for high-quality Kikuchi pattern acquisition. This synergistic preparation strategy successfully minimized lattice distortion artifacts, enabling precise investigation of dynamic recrystallization behavior and texture evolution during forging processing of AZ80 alloy.

### 2.2. Preparation of MAO and MCC Coatings

The MAO process is carried out using a single-pulse direct-current (DC) power supply in constant-current mode, made of Xi’an Flit Heat Treatment CO., Ltd. The experimental parameters are set as follows: a pulse current frequency of 500 Hz, a current density of 34 mA/cm^2^, a pulse width of 80 μs, a duty cycle of 15%, and a treatment duration of 10 min. These parameters were selected based on prior research findings to optimize the electrochemical reaction conditions during coating formation [26,27]. The MAO electrolyte is composed of 0.065 mol/L sodium silicate, 15 mol/L potassium fluoride, and 0.18 mol/L potassium hydroxide, with the pH adjusted to 13 using KOH solution to ensure high electrolyte conductivity and stability. A cooling system maintains the working temperature below 30 °C throughout the process. In the MAO setup, the magnesium alloy specimen acts as the anode, while a stainless steel plate serves as the cathode. The electric field generated between them induces micro-arc discharge, facilitating the formation of a dense and uniform oxide film on the magnesium alloy surface.

The MCC coating is formed by encapsulating the MAO-treated surface using electro-surge deposition followed by electrostatic spraying. The electrostatic spraying process (commonly referred to as E-paint) involves depositing finely atomized droplets of paint onto the substrate via electrostatic attraction. These droplets, composed of pigments and resin binders, are drawn to the substrate due to the opposing charges between the coating particles and the workpiece. This method ensures complete coverage, even on surfaces not directly exposed to the spray path. In this study, the E-paint consists of zinc phosphate as the primary pigment. Once cured, the zinc phosphate coating exhibits excellent corrosion resistance, further enhancing the protective properties of the composite layer.

### 2.3. Experimental Methods

#### 2.3.1. Salt Spray Accelerated Corrosion Test

To evaluate the corrosion behavior of AZ80 magnesium alloy under various forging conditions, salt spray accelerated corrosion tests were conducted for a duration of 35 days (840 h) as per the ASTM B117 standard [28]. During the initial corrosion stage, the microstructure and surface morphology of the alloys were examined using scanning electron microscopy (SEM), energy-dispersive X-ray spectroscopy (EDS), and optical imaging. Following the 35-day exposure, at least three replicate specimens from each alloy group were extracted for weight loss analysis. Prior to measurement, the corroded specimens were cleaned in the ASTM G1-03 standard solution [29], which was composed of 200 g/L CrO_3_, 10 g/L AgNO_3_, and 20 g/L Ba(NO_3_)_2_. The specimens were then dried and weighed using a precision electronic balance with a sensitivity of 0.1 mg. The mass loss and mass change per unit surface area were calculated to quantify the corrosion resistance. To evaluate the adhesion strength and corrosion protection effectiveness of the MCC coating, scratch testing was conducted following ASTM D1654 standard [30]. A standardized scratch (0.5 mm width) was introduced in the center of each MCC-coated specimen, penetrating through to the substrate. The corrosion resistance was subsequently assessed by measuring the corrosion creep perpendicular to the scratch direction. In this evaluation, superior corrosion resistance is indicated by minimal corrosion creep along both sides of the scratch.

#### 2.3.2. Stress Corrosion Cracking Test

**Figure 2:**
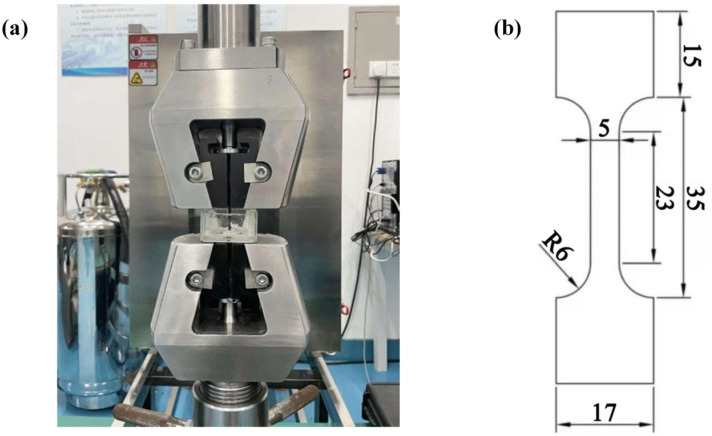
A corrosion cell mounted on a slow strain rate tensile (SSRT) testing machine for investigating the stress corrosion cracking (SCC) behavior of magnesium alloys in corrosive media. (**a**) Schematic diagram of the device; (**b**) Dimensions given in mm of specimens.

Slow strain rate tensile (SSRT) tests were performed on AZ80 magnesium alloy substrates, MAO, and MCC-coated specimens using a microcomputer-controlled universal testing machine with corroded vessels (Figure 2a). The specimens, with dimensions illustrated in Figure 2b, were tested at a constant strain rate of 10^−5^/s with a minimum loading rate of 0.012 mm/min. Prior to testing, each specimen was carefully aligned between the lower base and upper loading head to ensure proper load application. All tests were conducted in a 3.5 wt.% NaCl solution environment to evaluate the stress corrosion cracking (SCC) resistance. For each material condition, a minimum of three replicate tests were performed to ensure data reliability. Throughout testing, the applied load, fracture time, and displacement were continuously recorded. Tests were terminated upon complete specimen fracture and separation. Post-fracture analysis was conducted using SEM to examine the fracture surface morphology and determine the dominant failure mechanisms. The SCC susceptibility was evaluated based on the fracture characteristics and mechanical response during SSRT.

## 3. Results and Discussion

### 3.1. Microstructure

#### 3.1.1. Microstructure Evolution of AZ80 Magnesium Alloy

**Figure 3:**
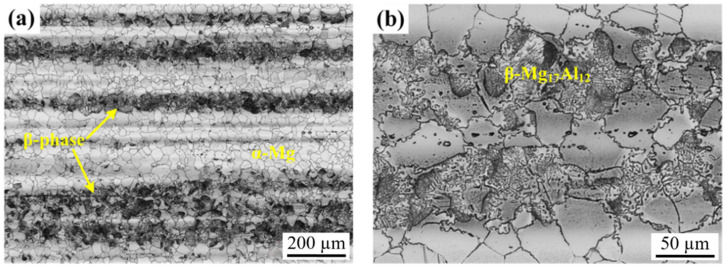
Microstructures at low magnification (**a**) and high magnification (**b**) of extruded AZ80E magnesium alloy at different magnifications.

Figure 3 displays the characteristic microstructures at low magnification (a) and high magnification (b) of extruded AZ80 magnesium alloy (AZ80E). The optical micrograph reveals a typical two-phase structure consisting of an α-Mg matrix with dispersed β-phase (Mg_17_Al_12_) intermetallic compounds. The relatively high aluminum content (8 wt.%) in this alloy promotes the formation of these β-phase particles, which exhibit a pronounced lamellar morphology preferentially aligned parallel to the extrusion direction.

**Figure 4:**
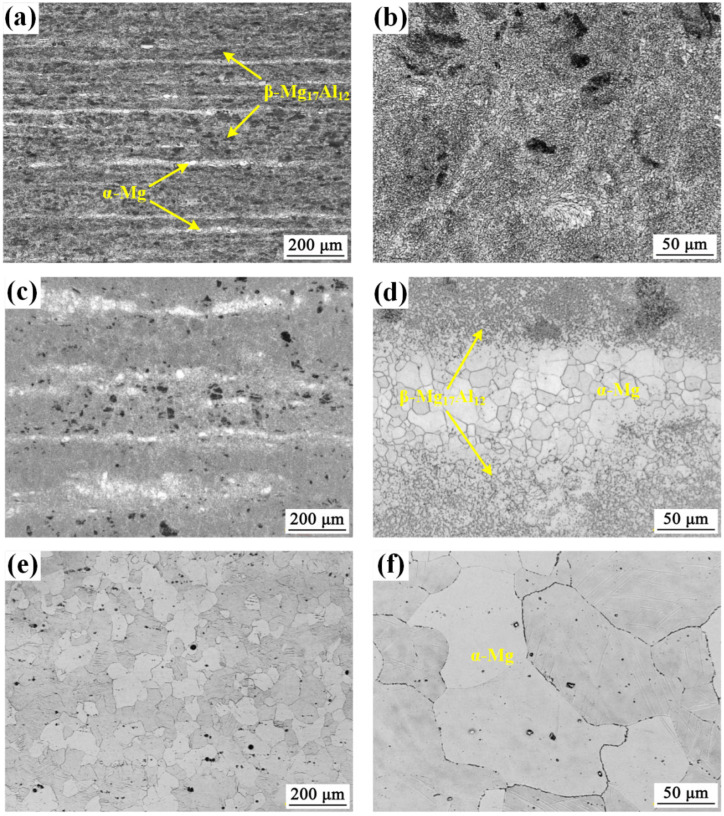
Microstructure of (**a**,**b**) AZ80F-250 alloy, (**c**,**d**) AZ80F-350 alloy, and (**e**,**f**) AZ80F-450 alloy, respectively.

Figure 4 presents the microstructural evolution of the AZ80E forged magnesium alloy processed at three different temperatures: 250 °C (AZ80F-250), 350 °C (AZ80F-350), and 450 °C (AZ80F-450). The AZ80F-250 alloy (Figure 4a,b) demonstrates significant microstructural refinement compared to the as-extruded AZ80E, featuring dynamically recrystallized (DRX) fine grains. The 250 °C forging process induces severe plastic deformation, resulting in both grain refinement and a more homogeneous distribution of the β-Mg_17_Al_12_ secondary phase particles. These findings are consistent with previous reports by Wang et al. [31]. At 350 °C (AZ80F-350, Figure 4c,d), the alloy maintains a fine-grained structure similar to AZ80F-250, though with slightly coarser DRX grains. However, one notable difference is the larger dynamic recrystallized (DRX) grain size observed in the AZ80F-350 alloy. This indicates that the forging process at 350 °C promotes the formation of slightly coarser grains compared to those formed at 250 °C. The microstructure undergoes a dramatic transformation at 450 °C (AZ80F-450, Figure 4e,f). The high processing temperature leads to complete dissolution of β-Mg_17_Al_12_ intermetallic compounds into the α-Mg matrix, producing a single-phase homogeneous structure. Concurrently, the DRX grain size in the AZ80F-450 alloy is relatively large, indicating that the elevated temperature facilitates the growth of larger grains during the forging process.

The microstructural evolution of AZ80 alloy demonstrates strong temperature dependence during forging, particularly regarding the dynamic interaction between β-Mg_17_Al_12_ phase transformation and dynamic recrystallization [24,32,33]. Due to the wide solidification range characteristic of Mg-Al alloys, aluminum progressively precipitates from the α-Mg matrix during forging, resulting in a typical microstructure comprising α-Mg solid solution and eutectic β-Mg_17_Al_12_ intermetallic phase. At relatively low forging temperature (AZ80F-250), lamellar β-Mg_17_Al_12_ precipitates are clearly observed within the supersaturated α-Mg matrix (Figure 4a), exhibiting a well-distributed morphology.

**Figure 5:**
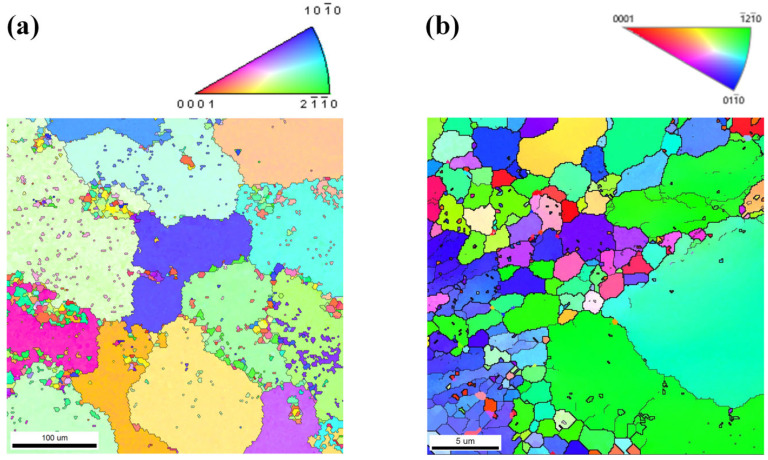
EBSD maps of (**a**) AZ80E alloy and (**b**) AZ80F-250 alloy.

Notably, the investigated specimens exhibit significant variations not only in β-Mg_17_Al_12_ phase distribution but also in DRX characteristics, including DRX degree, grain size distribution, and texture evolution. Figure 5 presents comparative EBSD analysis of AZ80E and AZ80F-250 alloys, revealing that forging temperature predominantly controls DRX grain size, while β-Mg_17_Al_12_ phase distribution provides secondary regulation [24,34]. Consistent with the optical micrographs in Figure 4, AZ80F-250 displays remarkably refined DRX grains (Figure 5b), with some regions even achieving nanoscale dimensions. The finely dispersed and continuous β-Mg_17_Al_12_ phase in this condition promotes homogeneous DRX nucleation and growth.

#### 3.1.2. Microstructure of MAO and MCC Coatings

**Figure 6:**
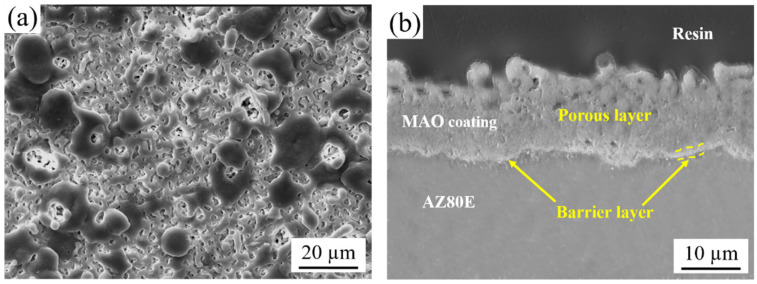
Morphologies of the MAO coating on AZ80E alloy: (**a**) surface and (**b**) cross-section areas.

MAO coatings were grown on AZ80E and AZ80F substrates. Figure 6 presents the characteristic surface and cross-sectional morphologies of the MAO coating formed on AZ80E. The coating surface exhibits a uniform distribution of micro-pores and oxide particles (Figure 6a), a typical morphological feature of MAO-treated magnesium alloys resulting from the rapid solidification of molten oxides and gas evolution during micro-arc discharges [9,35,36]. Cross-sectional analysis reveals that the MAO coating has a total thickness of approximately 15 μm, comprising a porous outer layer and a dense inner layer (~1 μm thick) adjacent to the substrate-coating interface (Figure 6b). This dense inner layer serves as an effective barrier against corrosive media penetration, significantly enhancing the corrosion protection of the magnesium alloy substrate. Furthermore, the MAO coating demonstrates excellent adhesion to the substrate through a metallurgical bond formed under high-temperature plasma conditions, thereby improving coating durability and long-term protective performance. Notably, SEM characterization indicated negligible differences in microstructure, thickness, and surface morphology between MAO coatings on extruded (AZ80E) and forged (AZ80F) alloys; therefore, only the AZ80E results are presented as representative. Phase analysis based on previous studies [27] confirms that the MAO coating primarily consists of MgO, MgF_2_, Mg_2_SiO_4_, and other complex silicate compounds.

**Figure 7:**
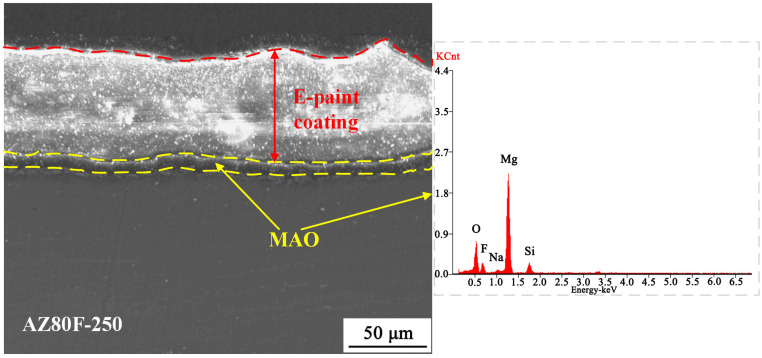
Cross-sectional morphology of the MCC coating on the AZ80F-250 substrate and EDS analysis of the MAO coating.

Figure 7 presents the cross-sectional micro-morphology of the AZ80F-250 wrought magnesium alloy after MCC coating treatment. As shown, two dense and uniform MCC coatings are formed on the alloy surface. The MAO coating, treated for 10 min, exhibits a thickness of approximately 15.5 μm. The average thickness of the E-paint layer by electrostatic spraying amounts to 63.2 μm. EDS analysis of the MAO coating within the MCC structure confirms that the E-paint coating does not alter the composition of the MAO layer. The bonding between the E-paint and MAO coatings is primarily physical, with strong integration achieved through the microporous structure of the MAO coating.

### 3.2. Accelerated Corrosion Behavior in Salt Spray

#### 3.2.1. Corrosion Behavior of AZ80 Alloys and MAO Coating

**Figure 8:**
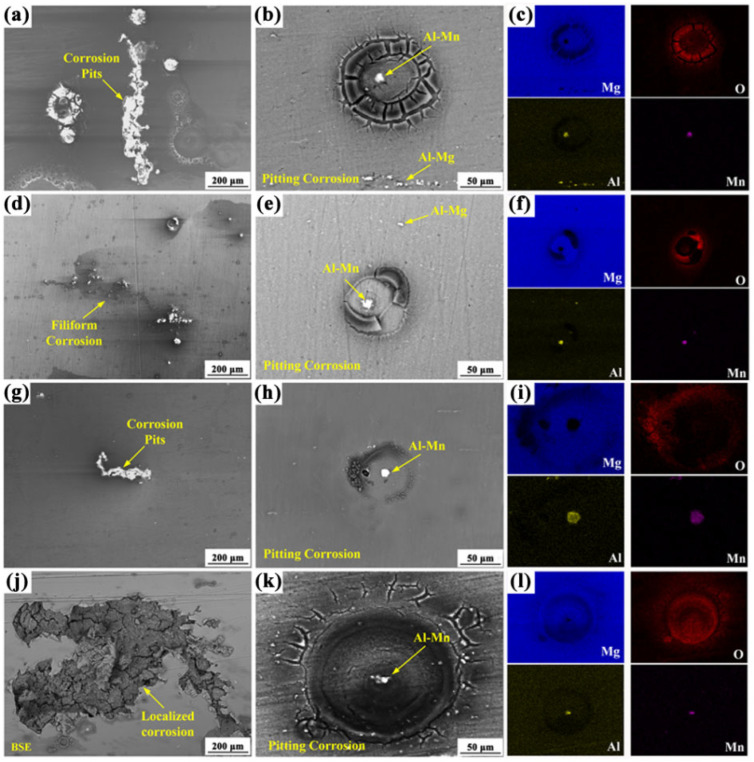
The surface corrosion morphologies of (**a**–**c**) AZ80E, (**d**–**f**) AZ80F-250, (**g**–**i**) AZ80F-350 and (**j**–**l**) AZ80F-450 alloys after continuous salt spray corrosion for 4 h, (**c**,**f**,**i**,**l**) are the EDX elemental mappings corresponding to (**b**,**e**,**h**,**k**), respectively.

Figure 8 displays the micro-corrosion morphologies and corresponding EDX elemental mapping analysis of different AZ80 matrix alloys following 4 h of accelerated corrosion. During initial corrosion stages, the inherent electrochemical heterogeneity of magnesium alloys leads to rapid microgalvanic coupling between the α-Mg matrix and β-phase (Mg_17_Al_12_) or other particles.

The AZ80 alloy exhibits distinct corrosion morphologies under different forging processes. As shown in Figure 8a,b, the AZ80E alloy surface displays corrosion pits aligned along the extrusion direction, characteristic of pitting corrosion. EDS analysis (Figure 8c) reveals that this pitting corrosion is primarily driven by microgalvanic coupling between the α-Mg matrix and Al-Mn particles, with β-Mg_17_Al_12_ phases also present on the surface. Quantitative EDS results show the Al-Mn particles have a composition of Mg 3.64 at.%, O 2.98 at.%, Al 58.26 at.%, and Mn 35.12 at.%, yielding an Al/Mn ratio of 1.66. This closely matches the stoichiometric ratio (1.6) of Al_8_Mn_5_ intermetallic compounds in magnesium alloys, consistent with previous reports [24,37,38]. Due to their higher corrosion potential compared to β-Mg_17_Al_12_, Al_8_Mn_5_ particles preferentially form microgalvanic couples with the α-Mg matrix, accelerating localized corrosion. The AZ80F-250 alloy (Figure 8d–f) exhibits multiple pitting sites with filamentous corrosion features distributed across the surface. Both Al_8_Mn_5_ particles (composition: Mg 4.58 at.%, O 0.68 at.%, Al 55.70 at.%, and Mn 39.04 at.%, and β-Mg_17_Al_12_ particles contribute to this pitting corrosion. For the AZ80F-350 alloy, corrosion pits also emerge on its surface (Figure 8g–i). The corrosion areas form along the extrusion direction and are smaller than those formed on the AZ80E surface, which is related to its microstructure. Al-Mn particles are also present on the matrix surface and cause pitting corrosion. The AZ80F-450 alloy (Figure 8j–l) shows irregular, large-scale corrosion pits, with EDS analysis confirming Al_8_Mn_5_ particles as the primary corrosion initiators.

In the aggressive Cl^-^ corrosive environment, corrosion of the AZ80 alloy preferentially initiates at interfaces between the α-Mg matrix and intermetallic compounds, with pitting nucleation predominantly occurring near Al_8_Mn_5_ particles during initial stages. All intermetallic compounds in magnesium alloys exhibit higher electrode potentials than the α-Mg matrix, with Al_8_Mn_5_ demonstrating particularly strong cathodic behavior compared to β-Mg_17_Al_12_. Notably, these Al_8_Mn_5_ particles maintain irregular, coarse morphologies across various extrusion and forging conditions due to their exceptional thermal stability within the 250 °C–450 °C processing range [38,39]. Consequently, the corrosion resistance of AZ80 magnesium alloys is fundamentally governed by their microstructural characteristics, including grain size variations and the spatial distribution of intermetallic phases.

After 4 h of corrosion, the AZ80F-450 alloy demonstrated the most severe localized corrosion among the four tested alloys (AZ80E, AZ80F-250, AZ80F-350, and AZ80F-450). AZ80F-450 had the largest grain size (Figure 4e,f). At the forging temperature of 450 °C, the grains grew to form coarse grains, and the second phase β-Mg_17_Al_12_ disappeared. The Al_8_Mn_5_ impurity possesses thermal stability. Therefore, AZ80F-450 alloy was highly susceptible to corrosion in corrosive media. The AZ80E alloy exhibited less corrosion damage than the AZ80F-450 alloy, with corrosion products aligned along the extrusion condition. This was mainly due to the distribution of the secondary phase in AZ80E alloy and the smaller grain size than that of AZ80F-450 alloy. Remarkably, the AZ80F-250 alloy displayed the best corrosion resistance among the series, showing only limited filamentous and pitting corrosion. This enhanced performance originated from its refined α-Mg grains and optimized β-Mg_17_Al_12_ phase distribution (Figure 4a,b and Figure 5b), where DRX produced abundant grain boundaries and a uniform dispersion of lamellar β-Mg_17_Al_12_ phases, consistent with established principles that fine-grained microstructures with secondary phase coverage enhance corrosion resistance [40,41]. As the forging temperature increased to 350 °C, the AZ80F-350 alloy exhibited grain growth relative to AZ80F-250, though maintaining smaller grains than AZ80E (Figure 4c,d). Accordingly, its corrosion resistance ranked between that of AZ80F-250 and AZ80E alloys.

**Figure 9:**
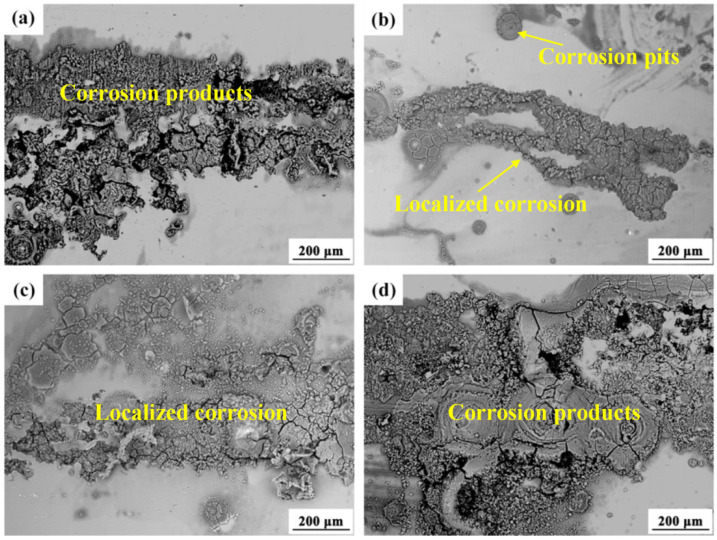
The surface corrosion morphologies of (**a**) AZ80E, (**b**) AZ80F-250, (**c**) AZ80F-350, and (**d**) AZ80F-450 alloys after continuous salt spray corrosion for 4 h.

With prolonged corrosion exposure, initial pitting corrosion develops into corrosion pits. The sustained activity of local galvanic cells facilitates rapid chloride ion (Cl^−^) accumulation within these pits, promoting localized corrosion propagation and pit expansion. Figure 9 presents the BSE images showing the corrosion morphology of four AZ80 alloys after 24 h of accelerated testing. All AZ80 alloy substrates exhibited significant localized corrosion after 24 h. The AZ80F-450 alloy showed particularly severe degradation, with corrosion products nearly covering its entire surface (Figure 9d), indicating poor corrosion resistance and high corrosion rates. In contrast, the AZ80E, AZ80F-250, and AZ80F-350 alloys displayed corrosion propagation primarily along the extrusion direction. Comparative analysis revealed more extensive continuous corrosion areas on the AZ80E (Figure 9a) and AZ80F-350 (Figure 9c) alloys relative to the AZ80F-250 alloy (Figure 9b).

**Figure 10:**
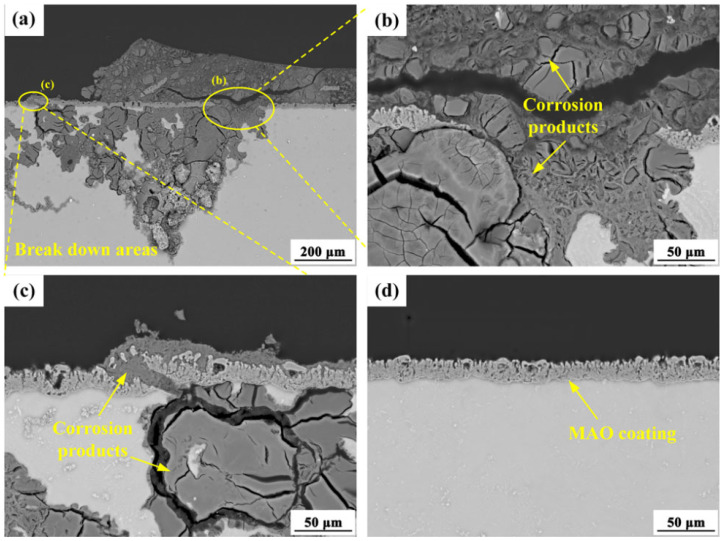
Cross-sectional morphology of the MAO coating on AZ80E alloy after 6 days of accelerated salt spray corrosion: (**a**–**c**) coating breakdown areas, (**d**) coating non-breakdown area.

Compared to the substrate alloys, the MAO-coated specimens exhibited a significantly reduced corrosion rate, confirming the exceptional protective capability of the hard ceramic MAO coating for magnesium alloys. The phase composition and chemical constituents of MAO coatings on magnesium alloys are predominantly determined by the MAO electrolyte formulation and the substrate’s chemical composition. As a result, when different forged AZ80 alloys undergo identical MAO treatment, they develop coatings with comparable thicknesses, microstructural characteristics, and corrosion protection performance. After a standardized 6-day salt spray accelerated corrosion test, localized corrosion was observed on all AZ80 alloy specimens, with corrosive attack penetrating the MAO coating. This behavior can be attributed to the infiltration of corrosive media through micro-pores and micro-cracks in the MAO coatings, followed by the diffusion of aggressive ions beneath the coating (Figure 10). Upon coating fracture initiation (Figure 10b,c), the corrosion rate of the MAO-coated specimens becomes primarily governed by the inherent corrosion resistance of the underlying AZ80 substrate. In contrast, regions where the coating remained intact (Figure 10d) exhibited no signs of corrosion degradation. Consequently, the corrosion resistance ranking of the MAO-coated specimens closely mirrors that of their respective AZ80 substrate alloy.

#### 3.2.2. Mass Loss Measurement

**Figure 11:**
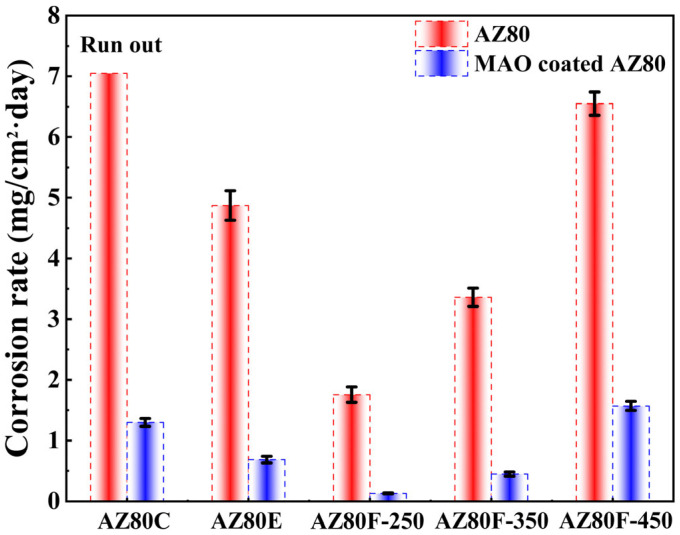
Corrosion rates of AZ80 magnesium alloy and MAO-coated specimens after 35 days of salt spray accelerated corrosion test under different forming processes.

Figure 11 presents the weight loss corrosion rates of AZ80 wrought magnesium alloys and MAO-coated specimens under different forging processes after 35 days of salt spray accelerated testing. Among all tested AZ80 alloys, the cast AZ80C (included from previous research [27] for comparison) demonstrates the highest corrosion rate and completely corrodes before completing the 35-day test period. This accelerated corrosion primarily results from severe micro-galvanic corrosion between α-Mg grains and second-phase precipitates, compounded by the presence of inclusions and porosity defects within the alloy. The corrosion resistance of magnesium alloys is significantly influenced by the distribution of the β-Mg_17_Al_12_ second phase. The phase exhibits a higher corrosion potential than the α-Mg matrix, enabling dual functionality: it can either act as a micro-cathode to accelerate α-Mg matrix corrosion or form a continuous protective network to inhibit matrix corrosion [42]. In AZ80F-250 and AZ80F-350 alloys, the fine and uniformly distributed second-phase particles create a nearly continuous corrosion barrier network, effectively reducing the overall corrosion rate. Conversely, in AZ80F-450 alloy, the dissolution of the second phase eliminates this protective network, leading to diminished corrosion resistance.

#### 3.2.3. Corrosion Behavior of MCC Coating

The MCC coating was prepared on AZ80F-250 wrought magnesium alloy. When subjected to identical salt spray accelerated corrosion testing conditions, both replicate specimens demonstrated exceptional corrosion resistance, showing virtually no signs of corrosion after 35 days of exposure. These results clearly indicate that the MCC coating provides outstanding protective performance for the magnesium alloy substrate.

**Figure 12:**
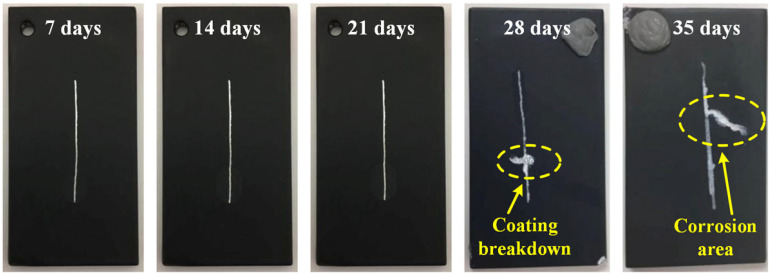
Macroscopic morphology of the MCC scratch coating on AZ80F-250 alloy after salt spray accelerated corrosion test for 7, 14, 21, 28, and 35 days.

To comprehensively evaluate the adhesion and corrosion protection properties of the MCC coating, scratch testing was performed in accordance with the ASTM D1654 standard [30]. A standardized 0.5 mm wide scratch was introduced on each MCC-coated specimen, reaching through to the substrate material. The corrosion resistance was quantitatively assessed by measuring the corrosion creep perpendicular to the scratch, where smaller creep distances indicate superior coating performance. Figure 12 presents the macroscopic evolution of scratch specimens subjected to salt spray accelerated corrosion testing over periods of 7, 14, 21, 28, and 35 days. Notably, the MCC coating demonstrated exceptional performance, with no observable general corrosion or rust creep along the scratch during the first 21 days of exposure. Initial signs of degradation became apparent at 28 days, manifesting as minor corrosion near the scratch and the onset of coating cracking. By 35 days, localized corrosion creep became evident along the cracked regions of the coating. These scratch test results confirm that MCC coating exhibits both excellent corrosion resistance and strong interfacial adhesion to the substrate. The delayed onset of corrosion creep and the limited progression of damage even after extended exposure highlight the coating’s robust protective capabilities.

The combination of MAO pre-treatment and subsequent electrophoretic (E-paint) coating via electrostatic spraying demonstrates exceptional corrosion resistance. When applied to MAO-coated substrates, the E-paint effectively seals micro-pores and micro-cracks in the MAO layer, creating a composite coating system with both interpenetrating and encapsulating characteristics. This process establishes strong interfacial adhesion between the E-paint and the MAO layer. Moreover, the electrostatic spraying technique enables the formation of a thick, dense protective barrier that provides comprehensive protection for both the underlying MAO coating and substrate material.

### 3.3. Stress Corrosion Cracking Behavior

**Figure 13:**
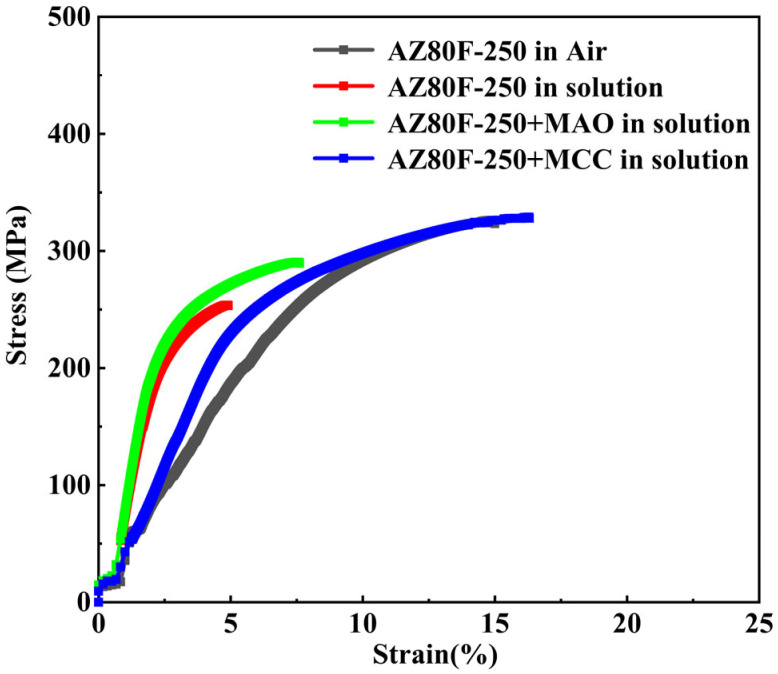
SSRT curves of AZ80F-250 and coated specimens in 3.5 wt.% NaCl corrosive solution.

The stress corrosion cracking (SCC) susceptibility of AZ80F-250 substrate and coated specimens was assessed via slow strain rate test (SSRT) in air and in a 3.5 wt.% NaCl corrosive solution at 10^−5^ s^−1^ strain rate. Figure 13 shows the stress-strain curves of uncoated, MAO-coated, and MCC-coated specimens in the corrosive medium, with the substrate’s behavior in air provided as a reference. In the corrosive environment, the AZ80F-250 substrate exhibited significant reductions in both tensile strength and ductility compared to its performance in air. This degradation stems from the alloy’s susceptibility to corrosion damage in the NaCl solution, which is further exacerbated by external stress application, leading to accelerated fracture. The MAO-coated specimens demonstrated moderate protection in the corrosive medium, showing improved tensile strength and ductility relative to the uncoated substrate. However, the inherent micro-pores and micro-cracks in the MAO coating served as initiation sites for stress-induced crack propagation, limiting its protective efficacy. In contrast, the MCC-coated specimens exhibited remarkable performance, with tensile strength and ductility values approaching those of the substrate tested in air. The composite coating effectively delayed specimen fracture time due to its ability to encapsulate and seal the microstructural defects present in the underlying MAO layer. This dense, impervious barrier maintained excellent protective capabilities even under applied stress, demonstrating superior SCC resistance. Based on the stress-strain curve, it can be seen from the perspective of elastic modulus that the main reason for the decrease in elastic modulus of magnesium alloys during slow strain rate tensile testing is the coupling effect of hysteresis, elastic relaxation, and early plastic deformation [11,22,43]. Compared with the elastic modulus of the AZ80F-250 substrate in air (3.3 GPa), the elastic modulus of the AZ80F-250 substrate (8 GPa), MAO (9.2 GPa), and MCC (4.6 GPa) specimens in the corrosive medium all increased. In corrosive media, the corrosion products of AZ80F-250 (Mg(OH)_2_, MgO) make their elastic modulus slightly higher than that of AZ80F-250 in air. However, the difference in elastic modulus between the corrosion products and the substrate will cause additional stress at the interface, which will accelerate crack propagation under dynamic loads. The MAO coating specimen also has the same reason, and the ceramic phase (MgO, MgF_2_, Mg_2_SiO_4_) of the MAO coating is more dense, the macroscopic elastic modulus is a little higher. Similarly, due to the difference in elastic modulus between the coating and substrate, as well as the defects in the coating itself, crack sources are formed, and the crack propagation is accelerated under stress. Due to the effect of the organic coating, the organic coating encapsulates the micro-pores and micro-cracks of the MAO coating, reducing its elastic modulus during the slow strain rate tensile process in 3.5 wt.% NaCl solution.

The SCC susceptibility of both substrate and coated specimens was quantitatively evaluated using the SCC susceptibility index (*I*_e_), calculated according to Equation (1) [44,45]. Table 1 presents the comparative stress-strain parameters for AZ80F-250 substrate and coated specimens tested in air versus 3.5 wt.% NaCl solution, from which the susceptibility indices were derived.

(1) $$I_{e}=\frac{\varepsilon_{air}-\varepsilon_{solution}}{\varepsilon_{air}}\times 100\%$$

where, *ε*_air_ and *ε*_solution_ are the elongations in air and solution, respectively. The tensile strength (UTS) and fracture time (*T*_f_) are also shown in Table 1. The UTS of the MCC-coated specimen increases by approximately 30%, and that of the MAO-coated specimen increases by approximately 14% in the corrosive medium. The decrease in *I*_e_ indicates an increase in SCC resistance. When *I*_e_ = 0, the experimental material is not sensitive to SCC. Compared with the single MAO coating (*I*_e_ 48.9 for the MAO-coated specimen), SCC resistance can be significantly improved by the MCC coating treatment in 3.5 wt.% NaCl corrosive medium.

**Figure 14:**
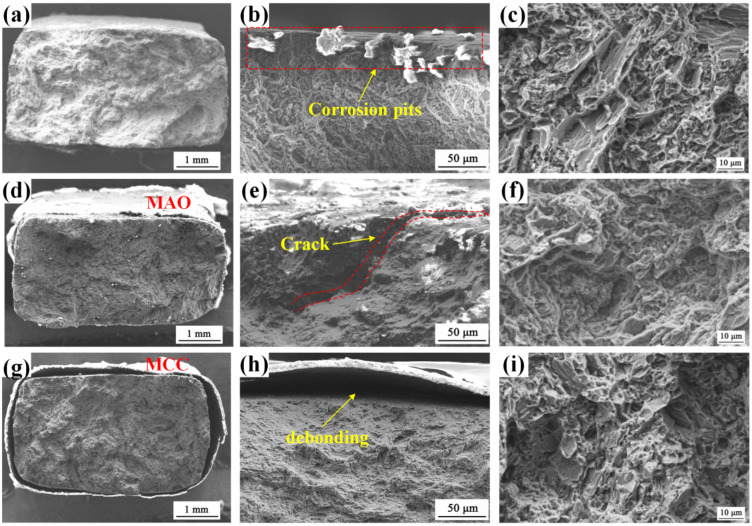
SEM images of the fracture morphologies of AZ80F-250, AZ80F-250 + MAO, and AZ80F-250 + MCC specimens in SSRT (**a**,**d**,**g**) overall, (**b**,**e**,**h**) crack initiation zone, and (**c**,**f**,**i**) final fracture zone, respectively.

Figure 14 presents the SCC characteristics of AZ80F-250 substrate, MAO-coated, and MCC-coated specimens in 3.5 wt.% NaCl solution. The AZ80F-250 substrate fracture surface exhibits distinct SCC features (Figure 14a), with multiple crack initiation sites originating from corrosion pits formed in the aggressive medium (Figure 14b). The final fracture zone displays characteristic brittle fracture morphology, featuring steep fan-shaped crack bands and a mixed intergranular-transgranular fracture mode, consistent with reported embrittlement behavior of wrought magnesium alloys [22,46,47]. The observed intergranular fracture may be attributed to electrochemical dissolution at grain boundaries induced by precipitates, which has been widely recognized as evidence of hydrogen-induced embrittlement in previous studies [48,49,50]. The ultimate fracture resulted from mechanical overload (Figure 14c). In contrast, MAO-coated specimens show less prominent SCC areas. While the MAO coating initially provides measurable protection to the AZ80F-250 substrate (Figure 14d), its inherent porosity and brittleness ultimately compromise its effectiveness. Surface micro-pores and micro-cracks serve as stress concentrators, promoting crack initiation under applied loads. This is evidenced by the presence of deep secondary cracks and extensive coating crazing (Figure 14e). As shown in Figure 14g, the MCC-coated specimen demonstrates superior performance, with primarily coating debonding observed and no significant corrosion morphology or crack initiation site. When combined with the stress-strain parameters in Table 1, these observations confirm the exceptional SCC resistance provided by the MCC coating. Notably, both MAO and SCC-coated specimens exhibit mixed transgranular-intergranular fracture characteristics in their SCC regions (Figure 14f,i), similar to the uncoated substrate, suggesting that the fundamental fracture mechanisms remain consistent across all specimens.

The defects of MAO coating mainly result from its formation mechanism: the thermal stress cracks caused by the difference in thermal expansion coefficients between the molten oxide and the substrate during cooling, as well as the additional stress generated at the interface between the coating and the substrate due to the difference in elastic modulus. Secondly, the insufficient local energy leads to the inclusion of unreacted oxide particles [9]. The methods to reduce the defects of MAO coating need to be comprehensively regulated from multiple aspects, such as process parameter optimization, electrolyte design, post-treatment technology, and composite coating strategy. Here, the MCC composite coating was selected. In the early stage, a dense and corrosion-resistant MAO coating was obtained by optimizing the process parameters [26,27]. By using an organic coating to seal the pores and provide a chemically inert barrier, combined with the low elastic modulus of the organic coating, the interface stress mismatch was alleviated.

### 3.4. Analysis of Corrosion Mechanism

**Figure 15:**
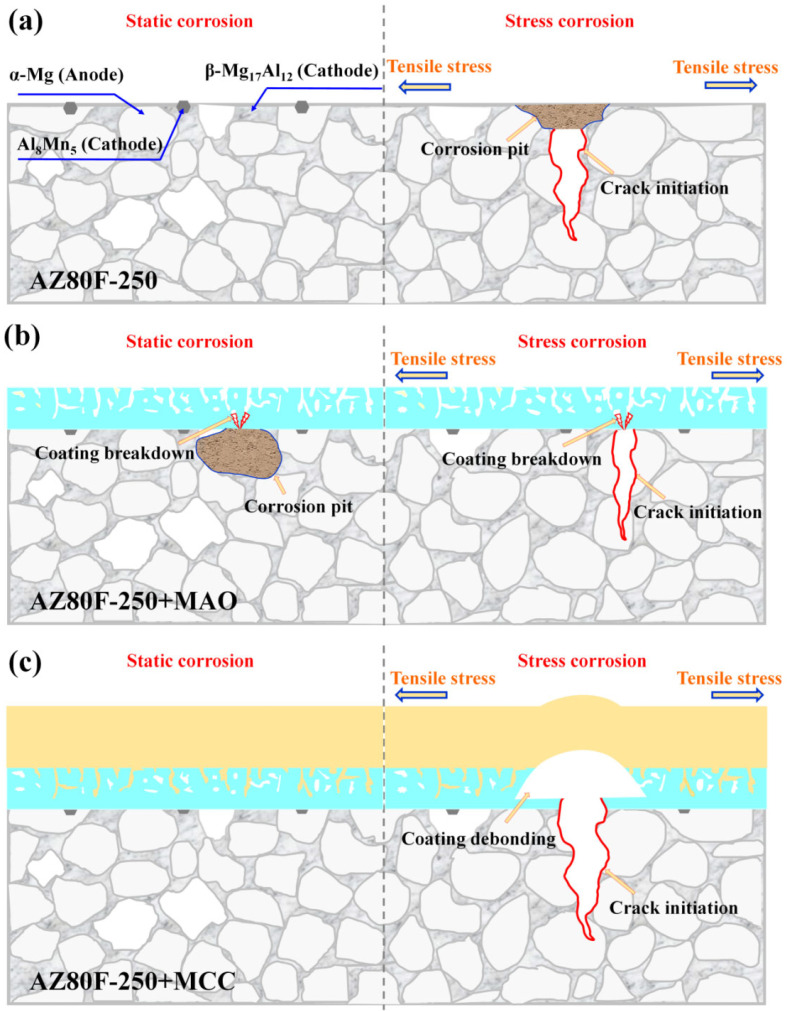
Schematic diagram of the static/stress corrosion mechanisms of (**a**) AZ80F-250 substrate, (**b**) MAO-coated specimen, and (**c**) MCC-coated specimen.

The static and stress corrosion protective performance of MAO and MCC coatings on AZ80F-250 alloy was systematically investigated. Under static corrosion conditions (Figure 15a), galvanic coupling between Al_8_Mn_5_ particles, β-Mg_17_Al_12_ phase, and the α-Mg matrix initiates pitting corrosion, which subsequently propagates into localized corrosion damage. While the MAO coating provides initial protection (Figure 15b), prolonged exposure allows corrosive media to penetrate through inherent micro-pores and micro-cracks, ultimately leading to coating breakdown and substrate corrosion. In contrast, the MCC coating demonstrates superior barrier properties, effectively protecting the substrate throughout the test duration.

Under SCC, uncoated specimens exhibit stress concentration at corrosion pits, serving as crack initiation sites that develop into mixed intergranular-transgranular fracture (Figure 15a). The MAO coating delays initial corrosion, but its porous structure facilitates crack nucleation when critical stress levels are attained (Figure 15b). As reported by Van Gaalen et al. [51] for WE43 alloy, MAO coatings produce more uniform corrosion fronts and shallower pits, consistent with our observations. However, once initiated, cracks propagate both inward to the subst31rate and outward through the coating [52], allowing solution penetration and eventual specimen failure. The MCC coating maintains excellent SCC resistance, with failure only occurring near the substrate’s ultimate tensile strength through coating debonding (Figure 15c). The synergistic combination of E-paint and MAO layers in the MCC system enhances both coating adhesion and corrosion protection. The MCC structure provides comprehensive defense against both static and stress corrosion mechanisms.

These findings establish fundamental design principles for magnesium alloy protective coatings and delineate formation pathways for stress-corrosion-resistant composite layers. The results provide valuable theoretical guidance and technical references for developing advanced protective coatings for magnesium alloy structural components under various service conditions. This design of the “inorganic-organic” gradient structure will continuously propel the development of magnesium alloy protective coatings and expand their application domains. It enables the coating system to concurrently satisfy contradictory performance demands, including conductivity/insulation, hydrophobicity/bio-compatibility, and wear resistance/friction reduction. Moreover, this design will facilitate the evolution of surface engineering in the direction of intelligent responsive coatings.

## 4. Conclusions

The corrosion behaviors of AZ80 wrought magnesium alloy substrate, MAO, and MCC coatings were investigated through static/stress corrosion tests. The failure behavior of the MAO coating and the protective mechanism of the MCC coating were also discussed. The following conclusions were drawn:

The magnesium alloy forged at 250 °C exhibits superior corrosion resistance relative to those forged at 350 °C, 450 °C, and the initial extruded AZ80 magnesium alloy (1.76 mg/cm^2^·day—salt spray corrosion rate). This enhanced performance stems from its characteristic fine-grained microstructure combined with an optimal network-like distribution of β-phase particles.

Under static corrosion conditions, both MAO (0.13 mg/cm^2^·day—salt spray corrosion rate) and MCC exhibit good corrosion resistance. Due to the inherent micro-pores and micro-cracks in the MAO coating, the coating breaks down as the corrosion time extends, leading to further corrosion of the substrate. The MCC coating, with its embedded and outer-coated organic layer, demonstrates even better corrosion resistance. Under stress corrosion conditions, the MAO coating defects serve as stress concentration sites that facilitate crack nucleation and propagation, ultimately leading to failure (the stress corrosion susceptibility of the substrate increased by 27%). The MCC coating demonstrates significantly superior protective performance. Particularly, it increased the stress corrosion susceptibility of the substrate by 100%. The MCC coating avoids the micro-defects of the MAO coating and the easy peeling of the E-paint organic coating, preventing the premature failure of magnesium alloys under static/stress corrosion conditions.

## Figures and Tables

**Table 1:** Stress-strain parameters of AZ80F-250 substrate and coating specimens in air and 3.5 wt.% NaCl solution.

Specimen	Corrosion Environment	Strain Rate/s^−1^	UTS/MPa	*T*_f_/h	*ε*/%	*I*_e_/%
AZ80F-250	Air	10^−5^	324	3.9	14.9	0
AZ80F-250	3.5 wt.% NaCl solution	10^−5^	253	1.3	4.9	67.1
AZ80F-250 + MAO		10^−5^	289	2.1	7.6	48.9
AZ80F-250 + MCC		10^−5^	328	4.0	16.3	−0.09

## Data Availability

The raw data supporting the conclusions of this article will be made available by the authors on request.
